# Cadmium Sulfide and Nickel Synergetic Co-catalysts Supported on Graphitic Carbon Nitride for Visible-Light-Driven Photocatalytic Hydrogen Evolution

**DOI:** 10.1038/srep22268

**Published:** 2016-02-29

**Authors:** Xinzheng Yue, Shasha Yi, Runwei Wang, Zongtao Zhang, Shilun Qiu

**Affiliations:** 1 State Key Laboratory of Inorganic Synthesis and Preparative Chemistry, College of Chemistry, Jilin University, Changchun 130012, P. R. China

## Abstract

Design and preparation of noble-metal-free photocatalysts is of great importance for photocatalytic water splitting harvesting solar energy. Here, we report the high visible-light-driven hydrogen evolution upon the hybrid photocatalyst system consisting of CdS nanocrystals and Ni@NiO nanoparticles grown on the surface of g-C_3_N_4_. The hybrid system shows a high H_2_-production rate of 1258.7 μmol h^−1^ g^−1^ in the presence of triethanolamine as a sacrificial electron donor under visible light irradiation. The synergetic catalytic mechanism has been studied and the results of photovoltaic and photoluminescence properties show that efficient electron transfer could be achieved from g-C_3_N_4_ to CdS nanocrystals and subsequently to Ni@NiO hybrid.

Graphitic carbon nitride (g-C_3_N_4_), a stable polymer photocatalyst, was found to be an attractive candidate for direct production of hydrogen by using solar energy in water, due to its high stability, nontoxicity, abundance and excellent optical property^1^,^2^. But the high photo-induced charge recombination rate and the lack of absorption above 460 nm of pristine g-C_3_N_4_ still make it unsatisfactory for practical applications. Then, it is necessary to load cocatalysts on pure g-C_3_N_4_ to achieve a high efficiency in spatial charge separation. As well known that, Pt has the largest work function and lowest overpotential, which is the best candidate cocatalyst for trapping electrons in H_2_-production photocatalytic reactions^3^,^4^. Taking into consideration of the cocatalysts on universality and low cost, the noble-metal based cocatalysts are too scarce and expensive to be used for large-scale energy production. The loading of cocatalyst not only can facilitate the charge separation but also can lower the activation energy or overpotential for the reactions. Therefore, pursuing a noble-metal free and highly active cocatalyst is highly desirable.

Cadmium sulfide (CdS), an attractive semiconductor sensitive to visible light, has been extensively studied for H_2_ evolution due to its narrow band gap and favorable band structure^5^,^6^,^7^. However, the sufficiently negative conduction band potential of CdS makes it photoinstability, which is usually due to photocorrosion (CdS + 2h^+^ → Cd^2+^ + S)^3^,^8^. Generally, construct semiconductor heterojunctions with controllable interface electronic structure undoubtedly to be an effective strategy to promote the separation of photo-induced carriers^9^,^10^. In that way, we employed CdS as H_2_ evolution synergetic catalyst on g-C_3_N_4_ to execute high hydrogen evolution activity and photostability^11^. Moreover, non-noble metal such as Co or Ni are also found to be efficient in photocatalytic H_2_ production loading on various semiconductors photocatalyst^12^,^13^. Agegnehu *et al.* found that ultrafine Ni nanoparticles loaded on graphene oxide (GO) sheets shows high photocatalytic properties for water splitting due to the easy transfer of photo-induced electrons from the GO photocatalyst to the Ni cocatalyst^12^.

To the best of our knowledge, there are no existing reports on the investigation of such a promising Ni@NiO/CdS/g-C_3_N_4_ system. Consequently, inspired by such these factors, the central goal of our current work is to improve the photocatalytic H_2_-production of g-C_3_N_4_ and explore the transfer of photo-induced charges in detail. Therefore, we report a Ni@NiO/CdS/g-C_3_N_4_ synergetic catalytic system, which investigated by presenting the excellent photocatalytic evolution properties and the enhanced photocatalytic activity mechanism. The broad range of light absorption character of CdS together with the efficient electron transfer from g-C_3_N_4_ to CdS nanoparticles and subsequently to Ni@NiO hybrid, attribute to the high photocatalytic H_2_ evolution activity of this composite photocatalytic system. The rate of H_2_ evolution of the optimized Ni@NiO/CdS/g-C_3_N_4_ is 486 times higher than that of pristine g-C_3_N_4_ and high stability can be achieved in these hybrid materials.

## Results

**Figure 1:**
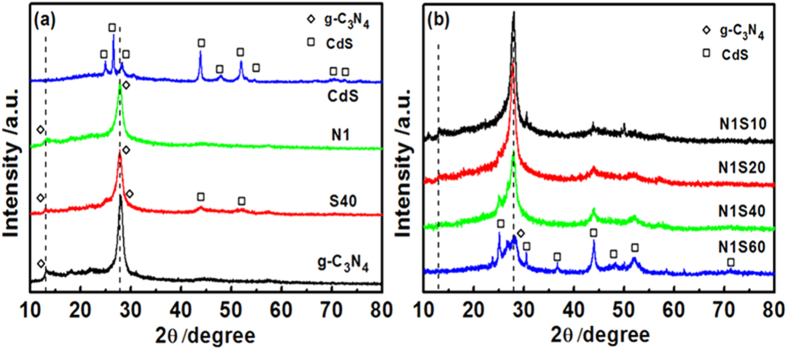
XRD patterns of (**a**) g-C_3_N_4_, S40, N1, and CdS; (**b**) N1S10, N1S20, N1S40 and 1SN60.

Fig. 1 shows the XRD pattern of the obtained graphitic-like layer structures of g-C_3_N_4_ with two distinct diffraction peaks^14^: the weak diffraction peak (100) centered at 13.1° was attributed to in-planar structural packing motif with a separation of 0.675 nm, and the strong one located at 27.4° corresponds to the (002) peak of the interlayer d-spacing of 0.326 nm^15^,^16^. For N1, no difference was found compared with pure g-C_3_N_4_, because that (i) Ni is not in the form of Ni^2+^ but possibility the metal Ni; (ii) the low loading percentages and low crystallinity of Ni. The CdS sample has hexagonal wurtzite structure in accordance with JCPDS No. 65-3414. The composite sample of S40 exhibited diffraction peaks corresponding to both g-C_3_N_4_ and CdS, reflecting the presence of two phases, which could be distinctly observed in Fig. 1b.

**Figure 2:**
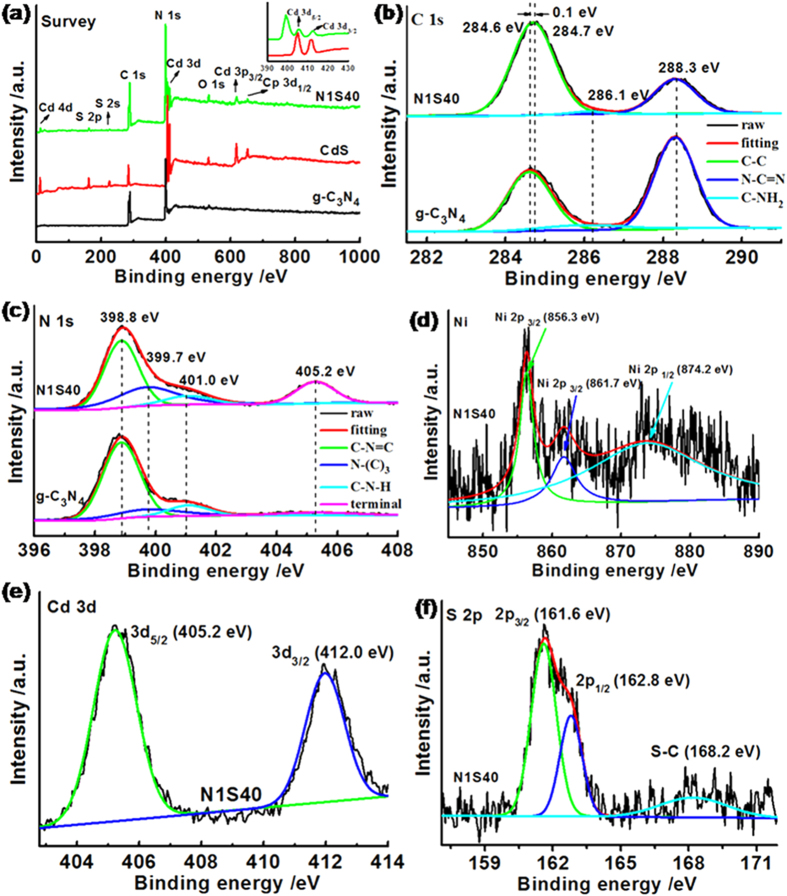
The XPS profiles of the obtained samples for (**a**) Survey, (**b**) C 1s, (**c**) N 1s, (**d**) Ni 2p, (**e**) Cd 3d, and (**f**) S 2p, respectively.

To precisely confirm the chemical composition and structure of the photocatalysts, X-ray photoelectron spectroscopy (XPS) was further undertaken in our work. In sample of N1S40, the typical C, N, Cd, S and O were observed, as in previous studies^15^. O is presumably originated from the surface absorbed H_2_O or CO_2_ molecules when urea under the high temperature pyrolysis in air^17^,^18^,^19^. The high resolution C 1s XPS spectra of pure g-C_3_N_4_ (Fig. 2b) show C-C, C-NH_2_, and N-C=N bonding at 284.6, 286.1 and 288.3 eV, respectively. Curiously, a slight C-C XPS peak shifts to higher binding energy, which can be ascribed to the chemical bonding between g-C_3_N_4_ and Ni/CdS. In Fig. 2c, the N 1s spectra can be fitted to four separate peaks at banding energy of 398.8, 399.7, 401.0 and 405.2 eV, respectively. The strong peak centering at 398.8 eV is identified as the sp^2^-hybridized N involved in triazine rings (C-N=C) and the peak at 399.7 eV regarded as the tertiary nitrogen N-(C)_3_ groups^14^,^19^. The weak peak at 401.0 eV indicates the presence of amino functional groups (C-N-H). Moreover, the last peak at 405.2 eV is attributed to terminal nitrate groups, charging effects, or 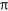 excitations^14^,^17^. Typically, metallic Ni nanoparticles are prepared by chemical reduction nickel precursor using NaBH_4_, but the XPS spectra peak to Ni^0^ is not found for the exposure of the sample to air to form a thin layer of NiO^12^,^20^. And the results indicate that the peak of Ni 2p_1/2_ at 874.2 eV are the divalent Ni^2+^ in NiO. Also, the peak of Ni 2p_3/2_ at 856.3 and 861.7 eV are the Ni^2+^ in Ni species^21^, which due to the fact that the Ni element can be easily oxidized by O_2_ in air. These phenomena imply that a thin NiO layer exists on the surface of Ni, the same as that in metallic Ni^22^. The high resolution Cd 3d XPS spectra of N1S40 (Fig. 2e) reveals the peaks of Cd 3d_5/2_ and Cd 3d_3/2_ located at 405.2 and 412.0 eV, which corresponded to the Cd^2+^ state^23^. Figure 2f shows the XPS signals of S 2p observed at 161.6 and 162.8 eV, as expected for the S^2−^ in CdS nanoparticles. It is worth noting that the peak around 168.2 eV can be assigned to the band between S and carbon substrate (S-C), suggesting the important interaction between g-C_3_N_4_ and CdS.

**Figure 3:**
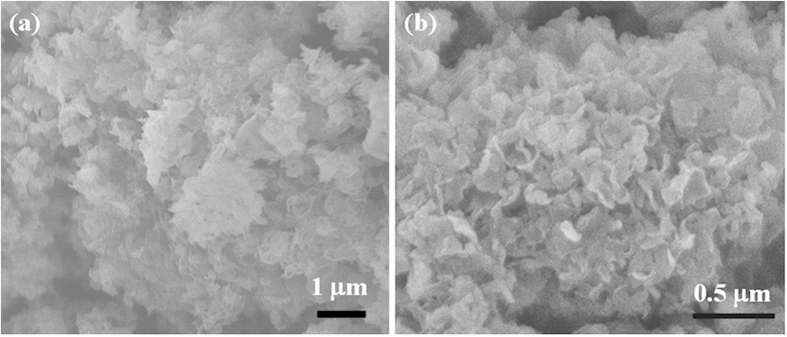
SEM images of (**a**) pure g-C_3_N_4_, and (**b**) N1S40 samples.

**Figure 4:**
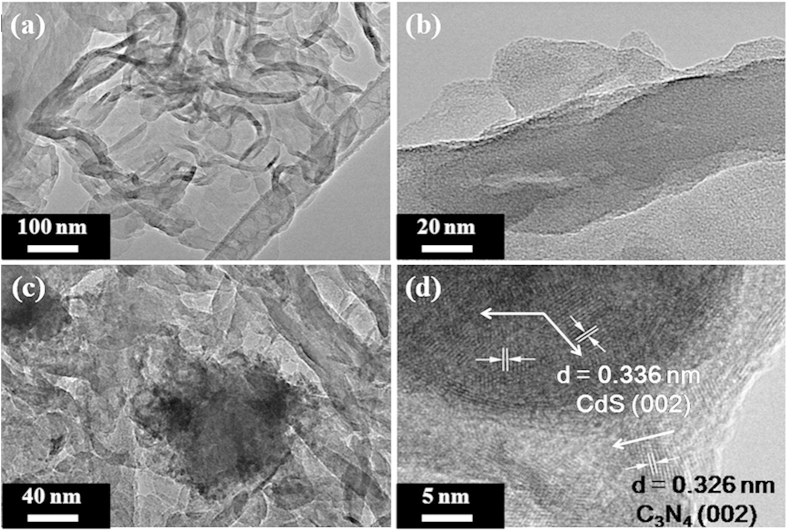
Typical TEM images of (**a,b**) pure g-C_3_N_4_ and (**c**) N1S40; (**d**) HRTEM image of N1S40.

The more detailed characterization of the morphologies and microstructures of the samples were based on SEM and TEM. Fig. 3 shows SEM images of (a) pure g-C_3_N_4_ and (b) N1S40 samples and the results present that both pristine g-C_3_N_4_ and N1S40 have a sheet structure with thin thickness. As illustrated in Fig. 4(a,b), the pristine g-C_3_N_4_ exhibits the morphology of stacking flat sheets with wrinkles and irregular shape, a two-dimensional structure with typical irregular porous. Fig. 4c gives direct evidence that CdS nanoparticles are firmly loaded on the surface of g-C_3_N_4_. The HRTEM image of N1S40 in Fig. 4d shows the crystallinity of CdS and g-C_3_N_4_ with an interplanar spacing of 0.336 nm and 0.326 nm, which are assigned to the (002) and (002) planes of the corroding phase, respectively.

### Optical absorption properties

**Figure 5:**
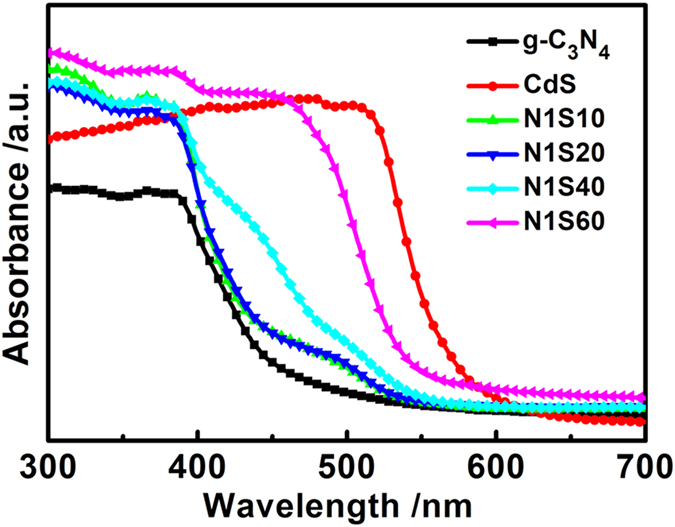
(**a**) UV-vis diffuse reflectance spectra (UV-vis DRS) of g-C_3_N_4_, CdS, N1S10, N1S20, N1S40 and N1S60 samples.

Fig. 5 shows the UV-vis DRS obtained to evaluate the optical absorption properties of the as-prepared products. The absorption edges of pure g-C_3_N_4_ and CdS are estimated to be 460 and 575 nm, which are correspond to the band gaps of 2.7 and 2.2 eV, respectively. After coupling with one species of Ni or CdS to g-C_3_N_4_, the composites of N1 or S40 shows the absorption edge at a higher wavelength with the absorbance intensity of them increased (Fig. S3). Meanwhile, with the CdS content increasing, remarkably enhanced absorbance in the visible region ranging from 450 to 700 nm was apparently observed in system of Ni/CdS/g-C_3_N_4_, which is because of their narrow band gap and deep color (Fig. 5)^24^. Moreover, this phenomenon gives a fact of intimate contact between light and the photocatalysts, which will facilitate the separation and transfer of photo-induced charge carriers in the hybrid structure^25^.

### Photocatalytic H_2_-production activity

**Figure 6:**
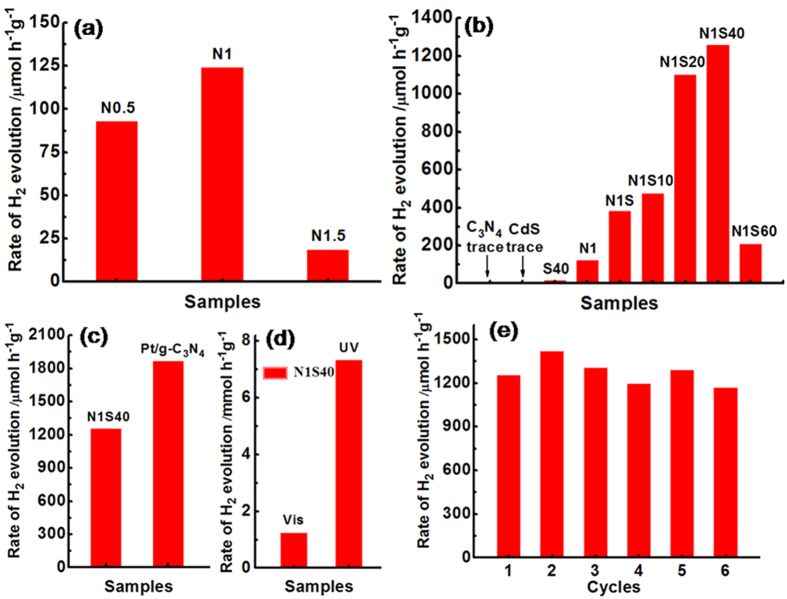
(**a**) Comparison of the photocatalytic H_2_ evolution activities of the Ni/g-C_3_N_4_ composites with different molar contents of Ni under visible-light irradiation of 4 h; (**b**) photocatalytic H_2_ production activities of different samples under visible-light irradiation of 4 h; (**c**) Comparison of the photocatalytic activity of the 1% Ni/CdS(40%)/g-C_3_N_4_ and 1% Pt/g-C_3_N_4_ for the H_2_ production under visible-light irradiation of 4 h; (**d**) Hydrogen evolution rates of 1% Ni/CdS(40%)/g-C_3_N_4_ both under UV and visible-light irradiation of 4 h and (**e**) Recycle test of Ni/CdS(40%)/g-C_3_N_4_ with every cycling time for 4 h. Light source: 300 W Xe lamp, λ > 420 nm. Reaction solution: 100 mL of aqueous solution containing 10 vol% of triethanolamine. Cat. 0.1 g.

Fig. 6 shows the photocatalytic hydrogen evolution activities of the aforementioned samples. From Fig. 6a,b, it can be seen that after loading slight Ni on the surface of g-C_3_N_4_, all Ni/g-C_3_N_4_ products show much higher photocatalytic activities than that of pure g-C_3_N_4_. Especially, the N1 sample shows the highest hydrogen evolution rate of 124.5 μmol h^−1^ g^−1^ for the recombination delay of electron-hole pairs in g-C_3_N_4_. Fig. 6b shows the H_2_-production performance of the CdS, S40, N1, N1S, N1S10, N1S20, N1S40 and N1S60 samples. It can be seen that pure CdS shows negligible activity because of recombination of electron-hole pairs. S40 (40% CdS/g-C_3_N_4_) also exhibits slight photocatalytic hydrogen production activities. That is to say, after loading either Ni or CdS, the photocatalytic hydrogen production activities of g-C_3_N_4_ are not significant enhanced. However, after addition two species of Ni and CdS into this system, the H_2_ evolution rate is improved remarkably, which may be credited to the existence of the synergetic effect between Ni and CdS. The optimal photocatalytic activity was achieved at 1% Ni and 40% CdS contents, whose high hydrogen-production rate reached to 1258.7 μmol h^−1^ g^−1^. Table S1 shows comparison of photocatalytic hydrogen evolution performance for Ni@NiO/CdS/g-C_3_N_4_ system with other photocatalysts. As shown in Fig. 6c, though 1% of Pt loading on g-C_3_N_4_ exhibited the higher photocatalytic activity toward H_2_ evolution than that of N1S40, we can say that the Ni/CdS also has superior cocatalytic activity on H_2_ evolution. Fig. 6d presents the hydrogen evolution rates of N1S40 under both UV and visible-light irradiation, and the results shows that the rate of H_2_ evolution over N1S40 reaches to a great value of 7.3 mmol h^−1^ g^−1^ under UV light irradiation.

To demonstrate the stability of Ni/CdS/g-C_3_N_4_ hybrid photocatalysts, recycling rest was performed and the results are shown in Fig. 6e. Almost no decrease H_2_-production rate is observed after six cycling irradiation of 24 h, indicating sufficient stability of this material for hydrogen generation. XRD and XPS analysis of the samples before and after the recycling experiment (Fig. S5 and S6) also illustrates the exceeding stability of our photocatalysts.

### Photovoltaic and photoluminescence properties

**Figure 7:**
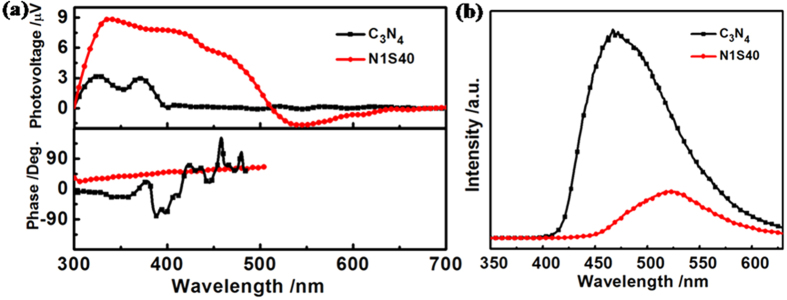
(**a**) SPV and the corresponding phase spectra and (**b**) PL spectra of g-C_3_N_4_ and N1S40.

To explore the separation and transfer process of the photo-induced charge carriers of the samples, the lock-in-based SPV measurements were carried out. Figure 7 shows the SPV spectra of g-C_3_N_4_ and N1S40. The pure g-C_3_N_4_ shows a weak signal while N1S40 presents much obvious response signal, which means that much more charge carriers were separated on N1S40 in spatially^26^,^27^. In addition, an interesting observation is that a negative response of N1S40 in the response region of 500–700 nm was found, which indicates that the electrons accumulate at the surface of the samples^28^. From the phase spectra in Fig. 7a, a less phase retardation with respect to −90° can be observed in comparing g-C_3_N_4_ with N1S40, and this phenomenon shows that the trend of photo-induced electrons moving to the outer surface of N1S40 sample^29^. That means much more photo-induced electrons have chances to take part in the photocatalytic H_2_ generation which results in increasing the photocatalytic H_2_ evolution rate of Ni@NiO/CdS/g-C_3_N_4_ system.

Fig. 7b shows the PL spectra of N1S40 and g-C_3_N_4_ excited at 325 nm. A strong PL emission peak is observed for g-C_3_N_4_, which can be attributed to the recombination of photo-induced electrons and holes. As a contrast, the PL emission peak intensity of N1S40 is much weaker than the g-C_3_N_4_, which confirms the high separation efficiency of the photo-induced charge carriers in composite of N1S40 and makes for the photocatalytic process.

In Fig. S7, a marked increase transient photocurrent response for N1S40 is observed as compared to pure g-C_3_N_4_ sample, which suggests that the mobility and separation of the photo-induced charge carriers is promoted by the synergetic cocatalysts of Ni/NiO/CdS supported on g-C_3_N_4_. In addition, after five light-on and -off cycles, the transient photocurrents of the two samples have no obvious decay, strongly once again indicating good stability of our photocatalysts.

## Discussion

It is well known that, to a great extent, the activity of a photocatalyst mostly depends on the separation and transfer of photo-induced electron-hole pairs. Then the band structures of CdS and g-C_3_N_4_ were revealed according to the previously reported works^5^,^6^. For CdS, the CB and VB positions are valued theoretically by the following empirical equations^5^,^6^: E_CB _= X − E_c _− 1/2E_g_ and E_VB_ = E_CB _+ E_g_, where X is the electronegativity for semiconductor; E_CB_ is the CB potential, E_VB_ is the VB potential; E_c_ is the energy of free electrons on the hydrogen scale (ca. 4.5 eV); and E_g_ is the band gap of the semiconductor. The X value of CdS is 5.18 eV^30^. So, the E_VB_ and E_CB_ of CdS are 1.78 and −0.42 eV, respectively. For g-C_3_N_4_, the E_VB_ and E_CB_ edge positions are 1.57 and −1.13 eV, respectively^31^.

**Figure 8:**
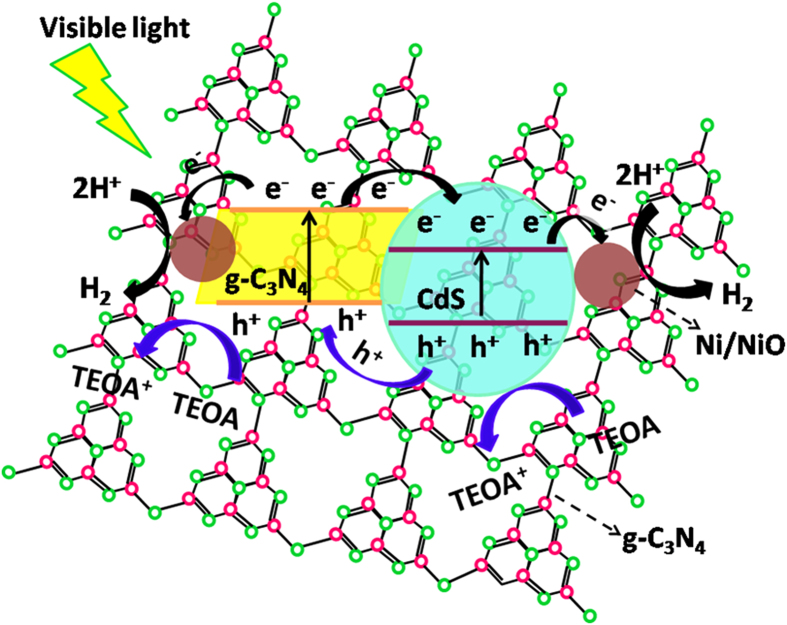
Schematic illustration of the mechanism for photocatalytic activity of Ni/CdS/g-C_3_N_4_.

Based on the above analyses, a postulated synergetic photocatalytic mechanism is proposed and depicted in Fig. 8. Under the visible light irradiation, the photo-induced charge transfer process would occur between CdS and g-C_3_N_4_ because of the inner electric field. More specifically, both CdS and g-C_3_N_4_ can be easily activated and generate electrons and holes under the irradiation of visible light. CB-electrons of g-C_3_N_4_ transfer to the CB of CdS and simultaneous VB-holes of CdS inject into the VB of g-C_3_N_4_. Besides, similar to the noble metal of Pt, Ni/NiO cocatalyst also has the ability of trapping electrons^12^. So the electrons will accumulate on the Ni/NiO reduction active sites for participating H_2_ evolution reaction, while holes to react with the TEOA in the aqueous solution. In this way, the efficient photo-induced electron-hole pairs lead to a significant enhancement of photocatalytic H_2_ production in the Ni/CdS/g-C_3_N_4_ composite system.

In summary, a hybrid nanophotocatalyst system with a 1258.7 μmol h^−1^ g^−1^ H_2_ evolution rate in triethanolamine solution has been achieved under vislble light irradiation. It is believed that the wide range of light absorption of CdS together with the efficient electron transfer from g-C_3_N_4_ to CdS nanoparticles and subsequently to Ni@NiO hybrid, attribute to the high photocatalytic H_2_ evolution activity of this composite photocatalytic system. This work not only shows a good strategy to enhance the photocatalytic H_2_-production activity of g-C_3_N_4_ by loading noble-metal-free cocatalysts of Ni@NiO/CdS, but also provides a new insight into the design and fabrication of other hybrid composite photocatalysts with high photocatalytic H_2_ evolution activity.

## Method

### Fabrication of Graphitic carbon nitride (g-C_3_N_4_)

g-C_3_N_4_ was synthesized thermally by heating urea (10 g) at 550 °C for 3 h with a heating rate of 4.6 °C min^−1^ under ambient pressure in air. Then, the as-obtained yellowish powder solid were collected and grinded to get the final sample.

### Fabrication of CdS/g-C_3_N_4_

A mixture of certain amount of Cd(NO_3_)_2_·4H_2_O, 0.083 g of thiourea and 0.5 g of g-C_3_N_4_ was dissolved in 25 mL of deionized water and ultrasonicated for 30 min. Then, the resulting solution was transferred into a 50 mL Teflon-lined stainless steel autoclave, sealed tightly, and heated at 180 °C for 12 h. Afterward the precipitates were washed several times with deionized water and ethanol, and then dried at 80 °C overnight. The molar ratios of CdS to g-C_3_N_4_ were 10%, 20%, 40%, and 60%, and the resulting samples were labeled as S10, S20, S40, and S60, respectively. Single phase CdS was also prepared using an identical procedure for comparison.

### Fabrication of Ni/g-C_3_N_4_, Ni/CdS, and Ni/CdS/g-C_3_N_4_

The Ni/g-C_3_N_4_, Ni/CdS, and Ni/CdS/g-C_3_N_4_ were prepared by a NaBH_4_ reduction method. Typically, g-C_3_N_4_ power (0.5 g) and Ni(NO_3_)_2_·6H_2_O (0.027 mM) were first ultrasonicated in deionized water (25 mL) for 5 min and subsequently stirred for 30 min. Then, NaBH_4_ solution (5 mL, 0.08 M) was gradually added to the above liquid, and the mixture was stirred for 5 min. After that, the obtained precipitates were washed and dried to get 0.5% Ni/g-C_3_N_4_. The deposition content of metal Ni can be varied by changing the amount of Ni(NO_3_)_2_·6H_2_O, and the final composites were marked as N0.5, N1, N1.5, correspondently. With the same method as above, we got the 1% Ni/CdS(10%)/g-C_3_N_4_, 1% Ni/CdS(20%)/g-C_3_N_4_, 1% Ni/CdS(40%)/g-C_3_N_4_, 1% Ni/CdS(60%)/g-C_3_N_4_, and 1% Ni/CdS, which were labeled as N1S10, N1S20, N1S40, N1S60, and N1S, respectively.

### Fabrication of Pt/g-C_3_N_4_

Typically, 0.5 g of g-C_3_N_4_ power was dispersed into 25 mL of deionized water containing 2.8 mL of H_2_PtCl_6_ aqueous solution (10 g/L), ultrasonicated for 5 min and subsequently stirred for 30 min. After that, NaBH_4_ solution (10 mL, 0.08 M) was quickly added to the above liquid, and the mixture was stirred for 30 min. After that, the obtained precipitates were washed and dried to get 1% Pt/g-C_3_N_4_.

### Characterization

The crystalline structure of the as-prepared sample was characterized by powder X-ray diffraction (XRD) with a Rigaku D/Max-2550 diffractometer using Cu Kα radiation (λ = 1.54056 Å) at 50 kV and 200 mA in the 2θ range of 10–80° at a scanning rate of 10° min^−1^. X-ray photoelectron spectroscopy (XPS) measurements were performed on a Thermo VG Scientific ESCALAB 250 spectrometer using monochromatized Al Kα excitation. The optical absorption spectra of the samples were measured on a UV-Vis-NIR spectrophotometer (Shimadzu UV-3600) detecting absorption over the range of 300–650 nm. SEM images were obtained on field emission scanning electron microscope (JSM-6700F, Japan). The transmission electron microscopy (TEM) was conducted on a Tecnai G2 S-Twin F20 TEM microscope (FEI Company). The element mappings were applied on a HITACHI SU-8020 transmission electron microscopy. N_2_ adsorption and desorption isotherms were carried out at 77 K using a Micrometrics ASAP 2020. The Brunauer-Emmett-Teller (BET) surface area was analyzed by a multipoint BET method using adsorption data in the relative pressure (P/P_0_) range of 0.05–0.25. Room temperature photoluminescence (PL) spectra with an excitation wavelength of 325 nm were measured on a FLUOROMAX-4.

The lock-in-based SPV spectroscopic measurement system consists of a source of monochromatic light, a sample cell, a computer, and a lock-in amplifier (SR830-D SP) with alight chopper (SR540). A low chopping frequency of 24 Hz was used. A 500 W xenon lamp (CHF-XM-500 W, Global Xenon Lamp Power) and a grating monochromator (Omni-5007, Zolix) provide monochromatic light. The samples were studied without further treatment duringthe SPV measurements, and the photovoltaic cell was a structure of fluorine tin oxide (FTO)-mica-sample-FTO. The system was calibrated by a DSI200 UV enhanced silicon detector to eliminate the possible phase shift which was not correlated to the SPV response, so that anyphase retardation reflected the kinetics of SPV response.

Photoelectrochemical measurements were performed with an electrochemical analyzer (CHI760E, Shanghai) in a three-electrode cell. The corresponding sample films on FTO used as the working electrode, Pt plate served as the counter electrode, and an Ag/AgCl (sat. KCl) acted as reference electrode. An aqueous solution of 0.5 M Na_2_SO_4_ was used as the electrolyte (pH = 7) and the voltage is 0.5 V versus Ag/AgCl. A 300 W xenon lamp was utilized as the simulated sunlight source.

### Photocatalytic H_2_-production

The photocatalytic hydrogen evolution experiments were performed with 0.1 g of photocatalyst suspended in a 100 mL solution containing 90 mL H_2_O and 10 mL triethanolamine, in a Pyrex glass reaction cell at ambient temperature and atmospheric pressure. A 300 W Xe lamp with cooling water (stabilize the temperature at 298 K) and a UV cutoff filter (≥ center wavelength 420 nm) was served as the visible-light source to trigger the photocatalytic reaction. Hydrogen gas evolution was analyzed using an online gas chromatograph (GC-8A, Shimadzu Co., Japan) equipped with an MS-5A column and a thermal conductivity detector (TCD). (see in Fig. S1).

## Additional Information

**How to cite this article**: Yue, X. *et al.* Cadmium Sulfide and Nickel Synergetic Co-catalysts Supported on Graphitic Carbon Nitride for Visible-Light-Driven Photocatalytic Hydrogen Evolution. *Sci. Rep.* **6**, 22268; doi: 10.1038/srep22268 (2016).

## Supplementary Material

Supplementary Information
