# Supplementary material for: Cadmium Sulfide and Nickel Synergetic Co-catalysts Supported on Graphitic Carbon Nitride for Visible-Light-Driven Photocatalytic Hydrogen Evolution
Source: Sci Rep. 2016 Feb 29;6:22268. doi: 10.1038/srep22268 (PMC4770297; doi:10.1038/srep22268)
Supplement: Supplementary Information [file srep22268-s1.doc]

Supplementary Information:

**Cadmium Sulfide and Nickel Synergetic Co-catalysts Supported on Graphitic Carbon Nitride for Visible-Light-Driven Photocatalytic Hydrogen Evolution**

Xinzheng Yue, Shasha Yi, Runwei Wang, Zongtao Zhang,* and Shilun Qiu

State Key Laboratory of Inorganic Synthesis and Preparative Chemistry, College of Chemistry, Jilin University, Changchun 130012, P. R. China.

*****Corresponding author: **Zongtao Zhang**

Tel: +86-431-85168115

Fax: +86-431-85168115

E-mail address: zzhang@jlu.edu.cn

1. **Hydrogen gas evolution equipment.**
2. **(a, c-h) Elemental mapping of N, C, O, Cd, S, and Ni elements in the N1S40 sample; (b) EDX spectrum of N1S40 sample.**
3. **UV-vis diffuse reflectance spectra (UV-vis DRS) of g-C_3_N_4_, CdS, S40 and N1, respectively.**
4. **(a) BET adsorption-desorption isotherms and (b) BJH pore size distribution of the g-C_3_N_4_, CdS, N1S20 and N1S40 samples.**
5. **XRD patterns of N1S40 before and after the recycling experiments.**
6. **The high-resolution XPS profiles of N1S40 after the recycling experiment for (a) Survey, (b) C 1s, (c) N 1s, (d) Ni 2p, (e) Cd 3d, and (f) S 2p, respectively.**
7. **Transient photocurrent responses of g-C_3_N_4_ and N1S40 samples in 0.5 M Na_2_SO_4_ solution under simulated solar light.**

**Figure S1. Hydrogen gas evolution equipment.**


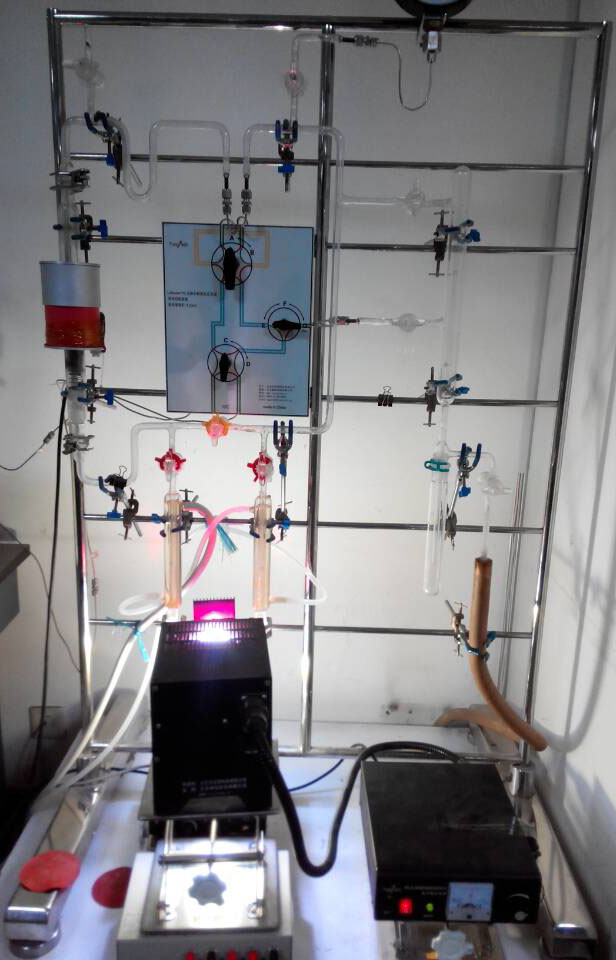


**Figure S2. (a, c-h) Elemental mapping of N, C, O, Cd, S, and Ni elements in the N1S40 sample; (b) EDX spectrum of N1S40 sample.**


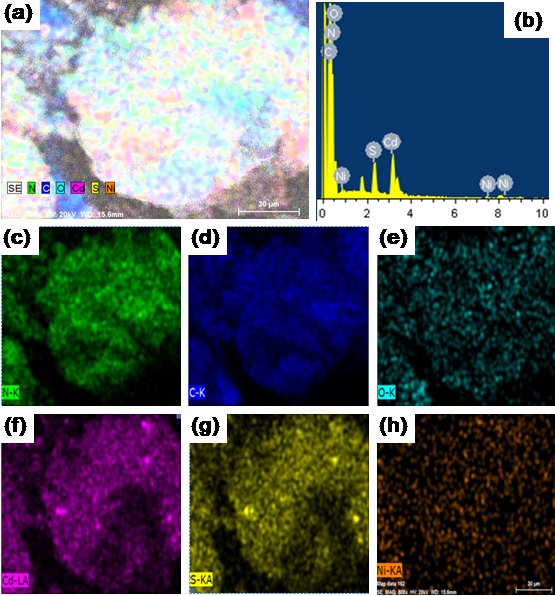


Figure S2(c-h) shows the elemental mapping of a space region in N1S40 and the results suggest that N, C, O, Cd, S, and Ni elements are all homogeneously distributed in the ternary composite system. Curiously, element of O is also discovered and uniformly dispersed in this structure (Figure S2e). This observation, together with the results of XPS (Figure 2d), indicates that NiO is authentic existence in our photocatalysts. Energy-dispersive X-ray spectroscopy (EDX) in Figure 5b is also used to confirm the presence of Ni, NiO and CdS. Then, it may be tempting to conclude that CdS and Ni@NiO are successfully combined with the g-C_3_N_4_.

**Figure S3. UV-vis diffuse reflectance spectra (UV-vis DRS) of g-C_3_N_4_, CdS, S40 and N1, respectively.**


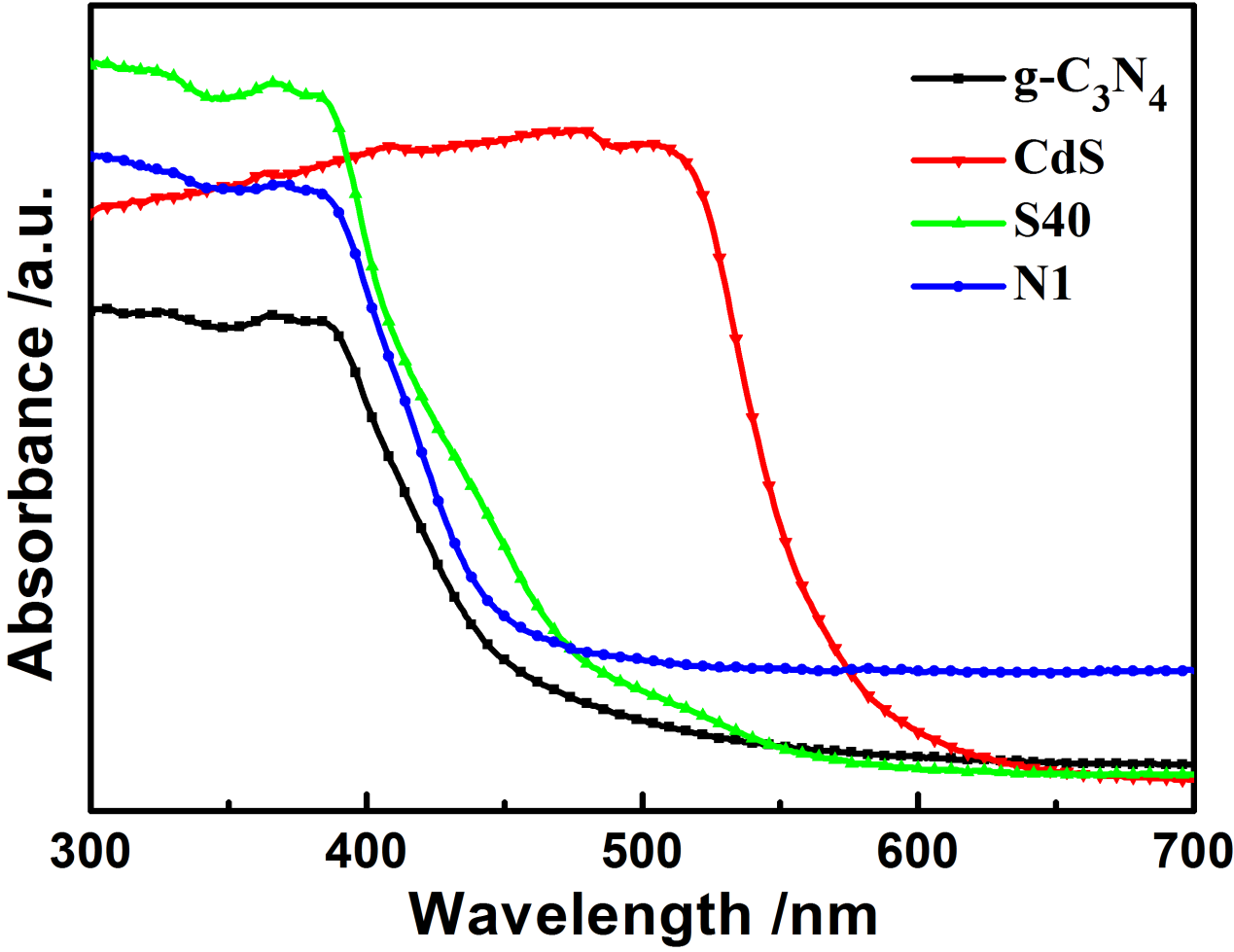


**Figure S4. (a) BET adsorption-desorption isotherms and (b) BJH pore size distribution of the g-C_3_N_4_, CdS, N1S20 and N1S40 samples.**


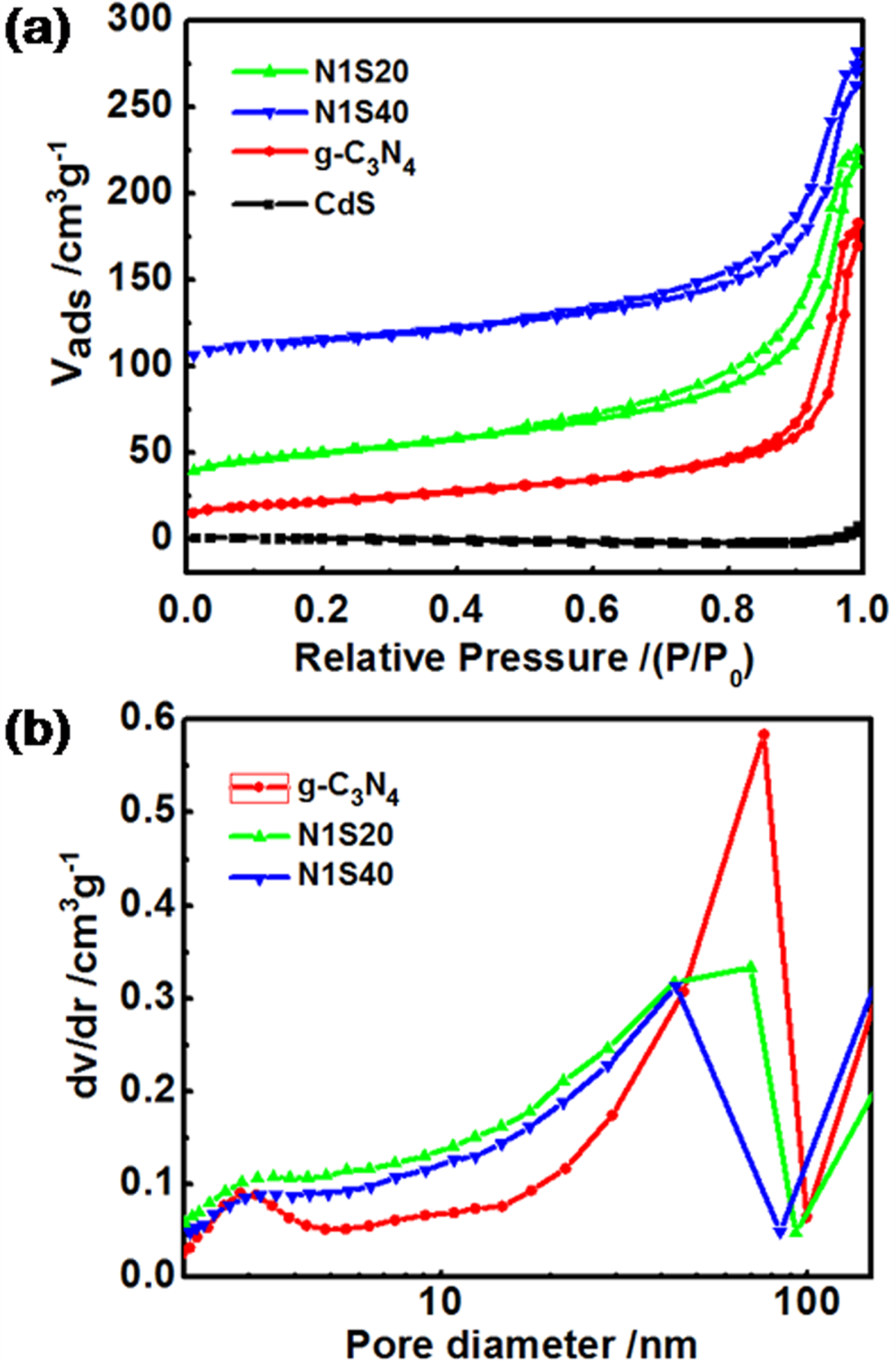


Nitrogen adsorption-desorption isotherms were performed to investigate the BET surface areas and porous structures of g-C_3_N_4_, CdS, and as-prepared Ni/CdS/g-C_3_N_4_. It can be seen that the isotherms of all present samples exhibit type IV behavior according to the classification of IUPAC, indicating the presence of mesopore (Figure S4). In addition, the BJH pore size distribution cuvers in Figure S4b all present some resemblance to type II isotherms, showing that the samples possess large macropores^1^. The BET surface areas of the N1S20 (101.91 m^2^ g^-1^) and N1S40 (89.68 m^2^ g^-1^) samples are higher than that of g-C_3_N_4_ (75.03 m^2^ g^-1^) sample. However, the pure CdS product has very little BET surface area with its value of 0.44 m^2^ g^-1^. Besides, it is interesting to find that the increased pore volumes are obtained from 0.20 cm^3^ g^-1^ for pure g-C_3_N_4_ to 0.26 cm^3^ g^-1^ for N1S20 and 0.25 cm^3^ g^-1^ for N1S40, indicating the valid separation behaviors of photo-induced charge carriers can be easier realized in the hybrid structure^2^.

**Figure S5. XRD patterns of N1S40 before and after the recycling experiments.**


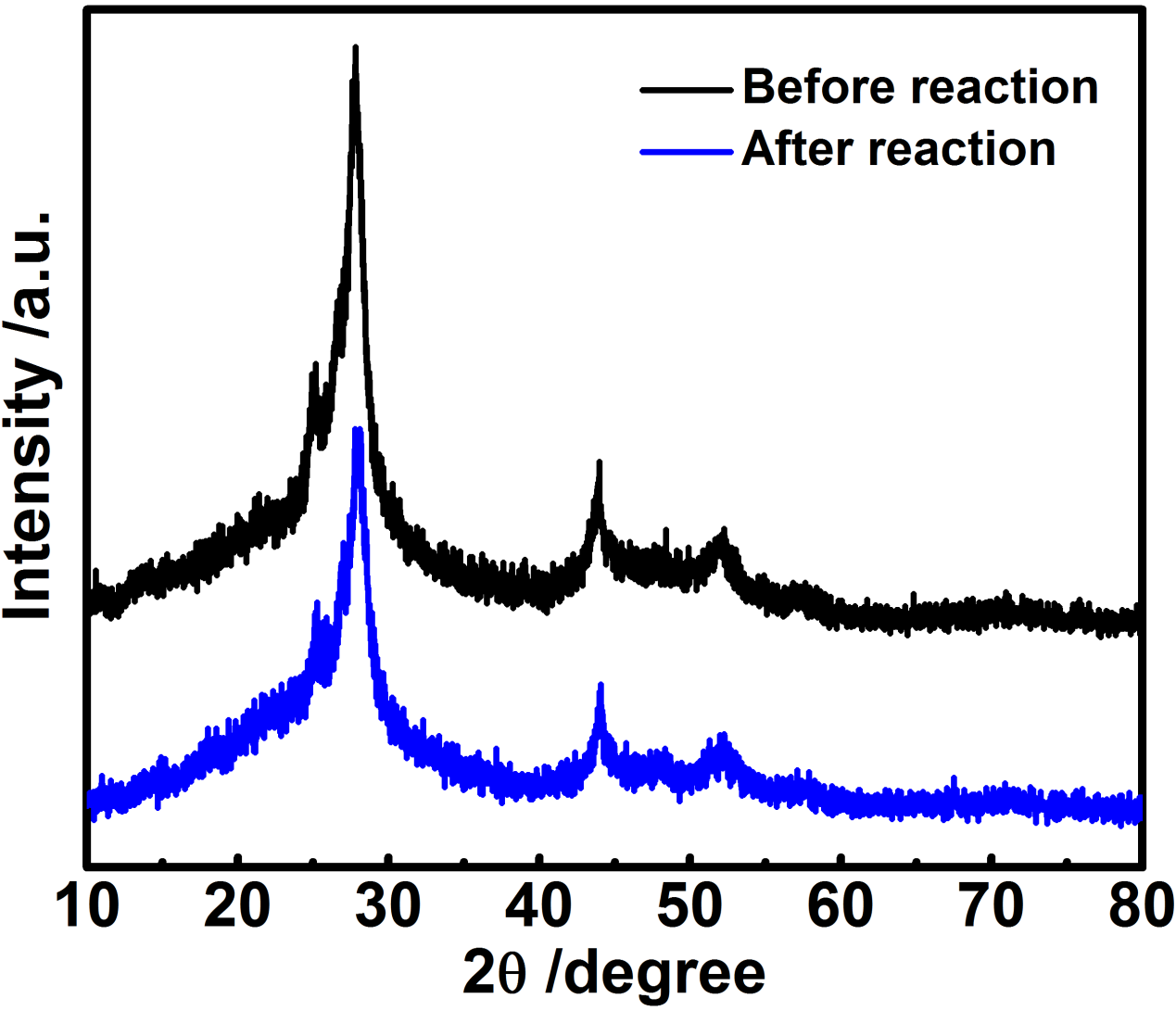


**Figure S6. The high-resolution XPS profiles of N1S40 after the recycling experiment for (a) Survey, (b) C 1s, (c) N 1s, (d) Ni 2p, (e) Cd 3d, and (f) S 2p, respectively.**


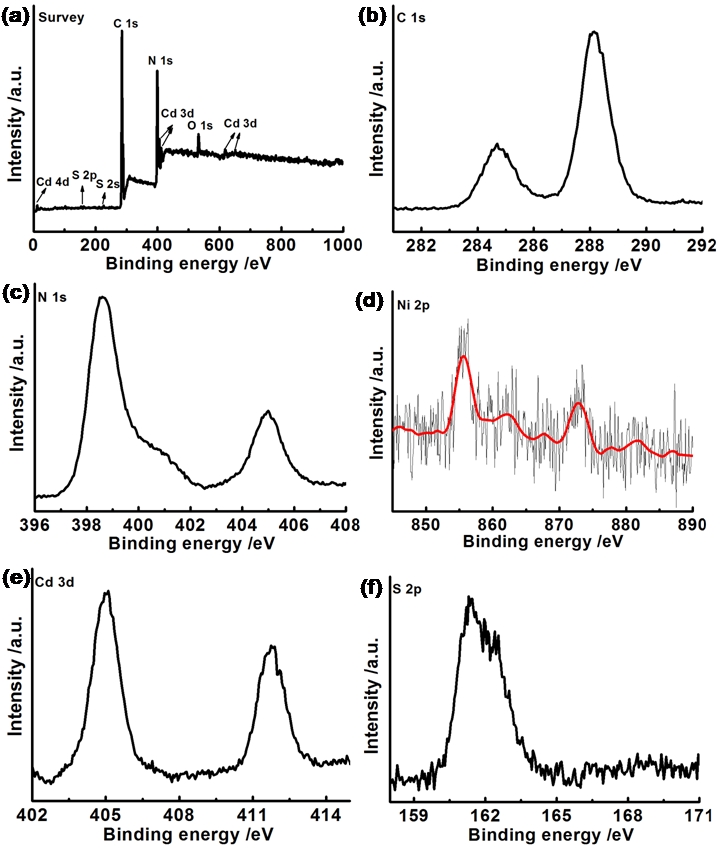


**Figure S7.** **Transient photocurrent responses of g-C_3_N_4_ and N1S40 samples in 0.5 M Na_2_SO_4_ solution under simulated solar light.**


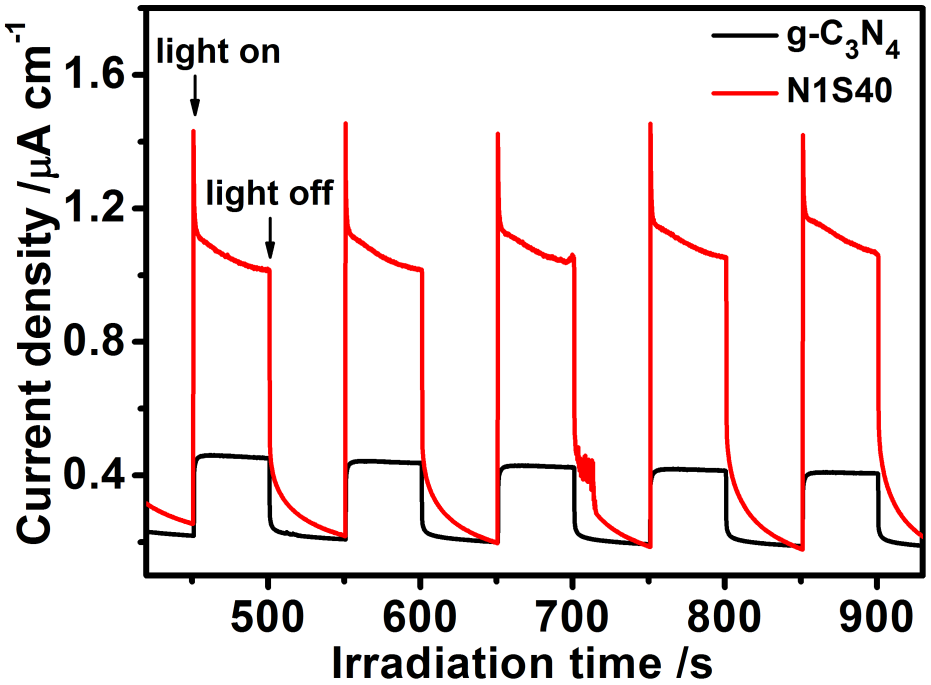


**Table S1. Comparison of photocatalytic hydrogen evolution performance for Ni@NiO/CdS/g-C_3_N_4_ system with other photocatalysts.**

| **Photocatalyst** | **Catalyst weight/g** | **Light source** | **H_2_** | **Ref.** | | | | | |  |
| --- | --- | --- | --- | --- | --- | --- | --- | --- | --- | --- |
| CdS/g-C_3_N_4_ | 0.1 | 300 Xe | 17.27 μmol h^-1^ | | | | | | 3 |  |
| ZnFe_2_O_4_/g-C_3_N_4_ | 0.1 | 350 Xe | 200.77  μmol h^-1^ g^-1^ | | | 4 | | | |  |
| Pt/CdS/g-C_3_N_4_ | 0.02 | 300 Xe | 601μmol h^-1^ | | 5 | | | | |  |
| NiS/g-C_3_N_4_ | 0.1 | 300 Xe | 48.2 μmol h^-1^ | | | | | | 6 |  |
| NiS/g-C_3_N_4_ | 0.1 | 300 Xe | 44.8 μmol h^-1^ | | | | 7 | | |  |
| MoS_2_/g-C_3_N_4_ | 0.02 | 300 Xe | 20.6 μmol h^-1^ | | | | | | 8 |  |
| NiS_2_/g-C_3_N_4_ | 0.01 | 300 Xe | 4.0 μmol h^-1^ | | 9 | | | | |  |
| WS_2_/g-C_3_N_4_ | 0.05 | 300 Xe | 11.0 μmol h^-1^ | | | | | | 10 |  |
| K-g-C_3_N_4_ | 0.1 | 300 Xe | 102.8  μmol h^-1^ | | 11 | | | | |  |
| Ni@NiO/CdS/g-C_3_N_4_ | 0.1 | 300 Xe | 1258.7  μmol h^-1^g^-1^ | | | | | This work | |  |

**References：**

1. Zhang, J. *et al.* Efficient Visible-Light Photocatalytic Hydrogen Evolution and Enhanced Photostability of Core/Shell CdS/g-C_3_N_4_ Nanowires. *ACS Appl. Mater. Interfaces* **5,** 10317-10324 (2013).

2. Kumar, S., Surendar, T., Kumar, B., Baruah, A., Shanker, V. Synthesis of Magnetically Separable and Recyclable g-C_3_N_4_-Fe_3_O_4_ Hybrid Nanocomposites with Enhanced Photocatalytic Performance under Visible-Light Irradiation. *J. Phys. Chem. C* **117,** 26135-26143 (2013).

# 3. Ge, L. *et al.* Synthesis and Efficient Visible Light Photocatalytic Hydrogen Evolution of Polymeric g-C_3_N_4_ Coupled with CdS Quantum Dots. *J. Phys. Chem. C* 116, 13708-13714 (2012).

4. Chen, J., Shen, S., Guo, P., Wu, P., Guo, L. Spatial engineering of photo-active sites on g-C_3_N_4_ for efficient solar hydrogen generation. *J. Mater. Chem. A* **2,** 4605-4612 (2014).

5. Zheng, D., Zhang, G., Wang, X. Integrating CdS quantum dots on hollow graphitic carbon nitride nanospheres for hydrogen evolution photocatalysis. *Appl. Catal. B: Environ.* **179**, 479-488 (2015).

6. Hong, J., Wang, Y., Wang, Y., Zhang, W., Xu, R., Noble-Metal-Free NiS/C_3_N_4_ for Efficient Photocatalytic Hydrogen Evolution from Water. *ChemSusChem* **6,** 2263-2268 (2013).

# 7. Chen, Z., Sun, P., Fan, B., Zhang, Z., Fang, X. In Situ Template-Free Ion-Exchange Process to Prepare Visible-Light-Active g-C_3_N_4_/NiS Hybrid Photocatalysts with Enhanced Hydrogen Evolution Activity. *J. Phys. Chem. C* 118, 7801-7807 (2014).

8. Hou, Y. *et al.* Layered Nanojunctions for Hydrogen-Evolution Catalysis. *Angew. Chem. Int. Ed.* **52**, 3621-3625 (2013).

9. Yin, L., Yuan, Y.-P., Cao, S.-W., Zhang, Z., Xue, C. Enhanced visible-light-driven photocatalytic hydrogen generation over g-C_3_N_4_ through loading the noble metal-free NiS_2_ cocatalyst**.** *RSC Advances* **4,** 6127-6132 (2014).

10. Hou, Y., Zhu, Y., Xu, Y., Wang, X. Photocatalytic hydrogen production over carbon nitride loaded with WS_2_ as cocatalyst under visible light. *Appl. Catal. B: Environ.* **156,** 122-127 (2014).

11. Wu, M., Yan, J.-M., Tang, X.-N., Zhao, M., Jiang, Q. Synthesis of Potassium-Modified Graphitic Carbon Nitride with High Photocatalytic Activity for Hydrogen Evolution. *Chemsuschem* **7,** 2654-2658 (2014).
